# Spontaneous Metabolic Regression in High‐Burden Follicular Lymphoma: A Case Report

**DOI:** 10.1155/crh/7545774

**Published:** 2026-03-06

**Authors:** Yotam Bronstein, Roy Vitkon, Lucille Hayman, Chava Perry

**Affiliations:** ^1^ Department of Hematology, Tel Aviv Sourasky Medical Center, Tel Aviv, Israel, tau.ac.il; ^2^ Gray Faculty of Medical & Health Sciences Tel Aviv University, Tel Aviv, Israel; ^3^ Institute of Pathology, Tel Aviv Sourasky Medical Center, Tel Aviv, Israel, tau.ac.il

**Keywords:** follicular lymphoma, high burden, spontaneous regression

## Abstract

Follicular lymphoma (FL) is an indolent but incurable B‐cell malignancy, typically requiring therapy in patients with high tumor burden. Although spontaneous regression has been described, its frequency and durability are uncertain and require longitudinal follow‐up. We report a 64‐year‐old man with high‐burden FL fulfilling GELF criteria who deferred treatment and subsequently demonstrated a marked spontaneous metabolic response on a follow‐up [^18^F]FDG PET/CT over a 6‐month follow‐up period. The metabolic response encompassed nodal, splenic, and serosal involvement, accompanied by resolution of symptoms and improvement in laboratory parameters. This case highlights the biological heterogeneity of FL and emphasizes the importance of individualized management and ongoing surveillance.

## 1. Introduction

Follicular lymphoma (FL) is the most common indolent B‐cell non‐Hodgkin lymphoma, representing 20%–25% of the cases in Western populations [1, 2]. It is commonly characterized by a protracted clinical course, repeated relapses, and a median survival exceeding 15 years. Despite its chronic nature, FL remains incurable with conventional therapy. Management is tailored to clinical presentation, disease burden, and patient preference.

Therapy is generally deferred in patients with low tumor burden and no significant symptoms. Observation, often termed “watch and wait,” has long been established as a safe initial strategy in this subgroup [3]. By contrast, patients who are symptomatic and those who meet the Groupe d’Etude des Lymphomes Folliculaires (GELF) criteria—such as bulky disease, cytopenias, or organ compromise—are recommended to receive systemic immunochemotherapy or, more recently, targeted therapies [4, 5].

Spontaneous regression of lymphoma is rare but has been most often described in indolent lymphomas, particularly FL and marginal zone lymphoma [6, 7]. Historical series estimated spontaneous regressions in 10%–20% of untreated FL cases, though typically incomplete and transient. With the increasing use of [^18^F]FDG PET/CT, metabolic changes can now be quantified, offering an objective means of documenting regression. However, published reports of [^18^F]FDG PET/CT‐documented regression remain limited.

We report a case of a patient with a high‐burden FL that fulfilled GELF criteria for treatment, yet demonstrated spontaneous metabolic regression on a follow‐up PET/CT during observation.

## 2. Case Presentation

A 64‐year‐old man presented with progressive lymphocytosis accompanied by constitutional and abdominal symptoms. He reported an unintentional 12 kg weight loss over approximately six months, as well as new‐onset abdominal distension and discomfort with a sensation of early satiety developing over the preceding weeks. He denied fever, night sweats, or other systemic symptoms. Past medical history included hypertension controlled on carvedilol and prediabetes, previously managed with metformin. No medication changes were made around the time of diagnosis and follow‐up. He was a nonsmoker with no significant alcohol use. His mother had a history of chronic lymphocytic leukemia.

On examination, the patient had generalized lymphadenopathy and palpable splenomegaly. Laboratory evaluation showed hemoglobin 10.6 g/dL, leukocytes 18.4 × 10^9^/L, absolute lymphocyte count 12.2 × 10^9^/L, and platelets 176 × 10^9^/L. Chemistry revealed creatinine 1.3 mg/dL (normal range: 0.7–1.3 mg/dL), alkaline phosphatase 165 U/L (normal range: 46–116 U/L), normal bilirubin, and LDH 411 U/L (normal range: 208–378 U/L).

Additional testing to exclude viral etiologies was unrevealing: HIV and hepatitis B/C were negative, and CMV serology and PCR were negative. EBV serology was consistent with prior infection (IgG positive and IgM negative), with EBV PCR negative.

Flow cytometry analysis of the peripheral blood demonstrated a monoclonal B‐cell population expressing CD19, CD20, CD22, CD200, CD79b, and CD10 with kappa light‐chain restriction, while negative for CD5, CD23, and FMC7.

Bone marrow biopsy revealed an infiltration by small cleaved lymphocytes accounting for about 40% of the bone marrow cellularity. Immunohistochemistry showed positivity for CD20, CD10, BCL2, and BCL6, with low Ki‐67 proliferation index. Cytogenetics confirmed the hallmark t(14; 18)(q32; q21) translocation.

**FIGURE 1:**
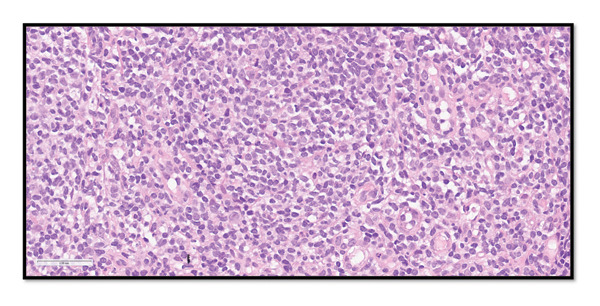
Left axillary lymph node core needle biopsy showing low‐grade (classic) follicular lymphoma. Hematoxylin & eosin (H&E), 40x. Left axillary lymph node core needle biopsy showing a nodular lymphoid infiltrate composed predominantly of small lymphoid cells. H&E, 40x.

**FIGURE 2:**
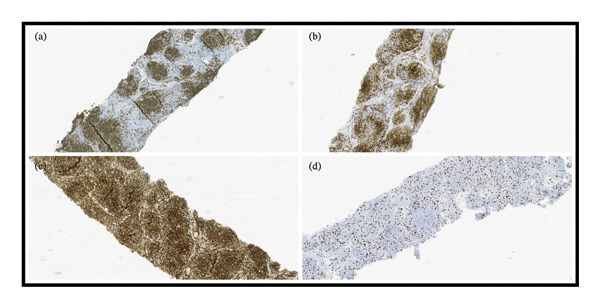
Immunohistochemistry of left axillary lymph node core needle biopsy supporting classic low‐grade follicular lymphoma. (a) CD20 highlights the neoplastic B‐cell population. (b) CD10 demonstrates germinal center marker expression in the neoplastic cells. (c) BCL2 shows positivity in the neoplastic follicles. (d) Ki‐67 demonstrates a proliferation pattern with a labeling index up to ∼15%.

Excisional biopsy of an axillary node confirmed the diagnosis of classic FL, WHO 5th edition, Grades 1‐2, without transformation (Figures 1 and 2).

Baseline [^18^F]FDG PET/CT (performed at diagnosis in February 2025) demonstrated widespread lymphadenopathy above and below the diaphragm (SUVmax up to 7.6), a bulky 7‐cm subcarinal mass, hepatosplenomegaly (liver 17.5 cm and spleen 16.2 cm), mild ascites, right pleural effusion, and diffuse intramedullary uptake. These findings were consistent with high tumor burden FL (Figure 3(a)).

FIGURE 3PET/CT scans showing spontaneous metabolic regression in high‐burden follicular lymphoma. Baseline PET/CT at diagnosis ((a) February 2025) demonstrated widespread fluorodeoxyglucose (FDG)‐avid lymphadenopathy above and below the diaphragm (SUVmax up to 7.6), bulky mediastinal disease, hepatosplenomegaly, ascites, and pleural effusion, consistent with high tumor burden follicular lymphoma. Follow‐up PET/CT performed six months later ((b) August 2025), without any systemic therapy, revealed marked regression of disease with substantial reduction in nodal size and metabolic activity (SUVmax ≤ 2.3), resolution of effusions, and improvement in hepatosplenomegaly, consistent with spontaneous metabolic regression.

**Figure:**
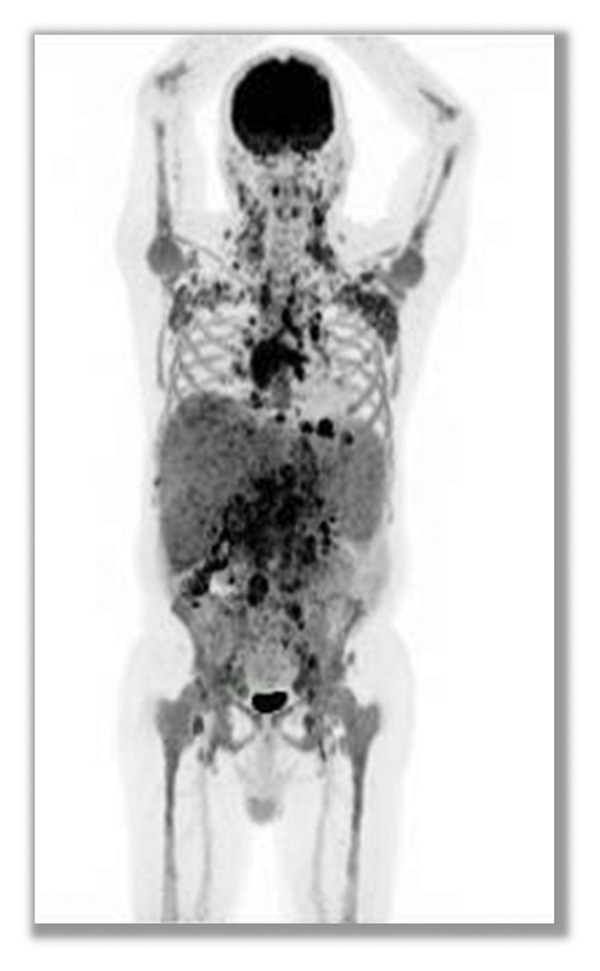
(a)

**Figure:**
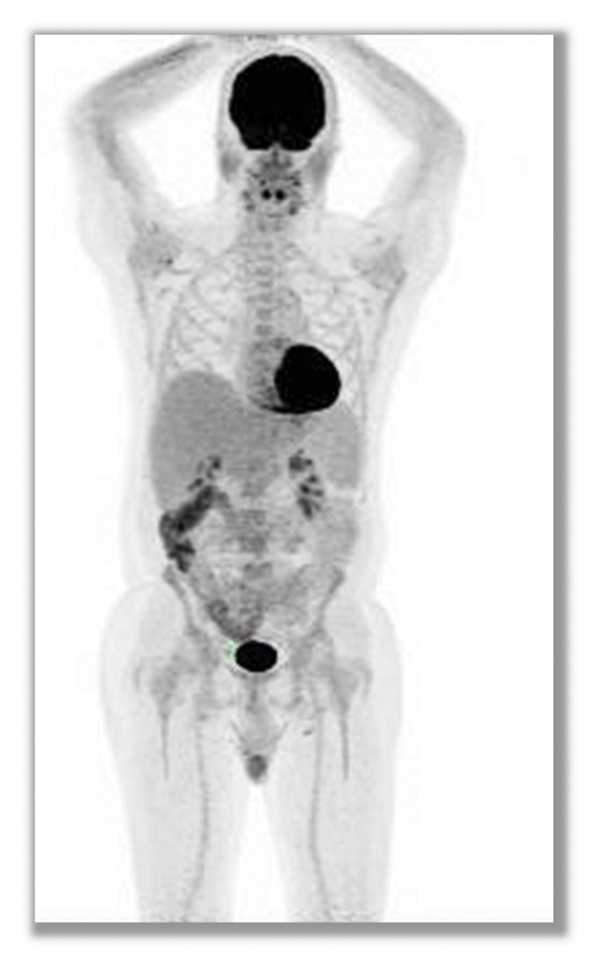
(b)

At that point, immunochemotherapy was recommended to the patient but despite his initial complaints and the high tumor burden, meeting the GELF criteria, the patient reported of improvement in his well‐being and preferred to defer treatment. A tight watch‐and‐wait approach was adopted. Over the subsequent weeks, his symptoms gradually improved, with decreasing abdominal distension and a slow, progressive weight gain. By three months following the baseline diagnosis, he had returned to his baseline weight and was without any systemic symptoms.

Follow‐up PET/CT that was performed six months later, in August 2025, showed marked regression: nodal masses decreased significantly in size, with maximum SUV reduced to 2.3 (Dueville score 3). The spleen decreased from 16.2 to 13.9 cm, hepatomegaly improved, and both ascites and pleural effusion resolved (Figure 3(b)). No new lesions were detected. Clinically, the patient remained asymptomatic, without B‐symptoms; hemoglobin improved to 11.9 g/dL, and LDH normalized.

This evolution was consistent with spontaneous metabolic regression.

Prior reports of spontaneous regressions suggested viral infections, such as EBV reactivation, as potential contributor to this phenomenon, yet the patient did not report of any illness during that time and PCR for EBV was negative.

## 3. Discussion

Here, we describe spontaneous regression in a patient with high burden FL.

Gattiker et al. first documented regression in 1980, describing disappearance of disease in untreated non‐Hodgkin lymphoma patients [6]. Subsequent long‐term series estimated that 10%–20% of untreated FL cases exhibited regression, though usually incomplete and temporary (“waxing and waning”) [7]. Most patients ultimately progressed and required systemic therapy.

Several mechanisms have been proposed. Immune‐mediated control is thought to be central, as FL is profoundly influenced by its microenvironment. T‐cell and NK‐cell activity may transiently suppress tumor proliferation, while loss of immune synapse function has been implicated in disease persistence [8]. Infectious triggers have also been observed, with viral or bacterial infections potentially inducing cytokine cascades that suppress tumor growth [9]. Another theory suggests that local trauma, including diagnostic biopsy, may stimulate inflammatory responses that extend beyond the biopsy site, provoking systemic regression [10]. Finally, molecular studies reveal heterogeneity in FL biology, with certain genetic and microenvironmental features possibly predisposing to variable natural histories [11]. In the present case, however, there was no clinical evidence of a preceding infection (the patient denied febrile illness) and no reported trauma during the relevant period, making these triggers less likely contributors to the observed regression.

What distinguishes the present case from previous reported cases is the documentation of regression in a patient with clearly defined high tumor burden, confirmed by metabolic imaging. While spontaneous regression has been reported in low‐burden disease, examples in patients meeting GELF criteria are rare. PET/CT not only confirmed anatomical shrinkage but also demonstrated reduced FDG avidity across nodal and extranodal compartments, providing strong evidence of true biological regression rather than sampling or measurement error.

Clinically, this case reinforces the principle that FL should be managed with flexibility, balancing disease biology, clinical presentation, and patient preference. While systemic therapy remains standard care for high‐burden disease, our patient’s course suggests that watchful observation can be safe in carefully selected individuals. Nonetheless, regression is usually transient, and continued vigilance is warranted, as relapse is expected in the majority of cases.

This report adds to the limited literature on spontaneous metabolic regression in FL and emphasizes the value of PET/CT in objectively documenting such events.

## 4. Conclusion

Our case of spontaneous partial regression in high‐burden disease underscores the biological heterogeneity of FL. Recognition of this rare phenomenon should encourage further investigation into the immunologic and microenvironmental mechanisms underlying regression, which may ultimately inform novel therapeutic strategies harnessing endogenous antitumor responses.

## Author Contributions

Yotam Bronstein initiated the study and wrote the manuscript, Lucille Hayman provided the pathology materials, and Roy Vitkon and Chava Perry reviewed the manuscript.

## Funding

This study received no external funding.

## Ethics Statement

The study was approved by the local ethics committee (IRB 17‐0733).

## Consent

The patient provided consent for publication, and all identifying details were removed to protect confidentiality in accordance with ethical guidelines.

## Conflicts of Interest

The authors declare no conflicts of interest.

## Data Availability

The data that support the findings of this study are available on request from the corresponding author. The data are not publicly available due to privacy or ethical restrictions.
